# Target Definition in Salvage Radiotherapy for Recurrent Prostate Cancer: The Role of Advanced Molecular Imaging

**DOI:** 10.3389/fonc.2016.00073

**Published:** 2016-03-31

**Authors:** Gaël Amzalag, Olivier Rager, Claire Tabouret-Viaud, Michael Wissmeyer, Electra Sfakianaki, Thomas de Perrot, Osman Ratib, Raymond Miralbell, Giampiero Giovacchini, Valentina Garibotto, Thomas Zilli

**Affiliations:** ^1^Division of Nuclear Medicine, Hospital of Neuchâtel, Neuchâtel, Switzerland; ^2^Division of Nuclear Medicine and Molecular Imaging, Geneva University Hospital, Geneva, Switzerland; ^3^Division of Radiology, Geneva University Hospital, Geneva, Switzerland; ^4^Faculty of Medicine, Geneva University, Geneva, Switzerland; ^5^Division of Radiation-Oncology, Geneva University Hospital, Geneva, Switzerland; ^6^Department of Radiology and Nuclear Medicine, Stadtspital Triemli, Zurich, Switzerland

**Keywords:** prostate cancer, PET, MRI, salvage radiotherapy, choline, PSMA

## Abstract

Salvage radiotherapy (SRT) represents the main treatment option for relapsing prostate cancer in patients after radical prostatectomy. Several open questions remain unanswered in terms of target volumes definition and delivered doses for SRT: the effective dose necessary to achieve biochemical control in the SRT setting may be different if the tumor recurrence is micro- or macroscopic. At the same time, irradiation of only the prostatic bed or of the whole pelvis will depend on the localization of the recurrence, local or locoregional. In the “theragnostic imaging” era, molecular imaging using positron emission tomography (PET) constitutes a useful tool for clinicians to define the site of the recurrence, the extent of disease, and individualize salvage treatments. The best option currently available in clinical routine is the combination of radiolabeled choline PET imaging and multiparametric magnetic resonance imaging (MRI), associating the nodal and distant metastases identification based on PET with the local assessment by MRI. A new generation of targeted tracers, namely, prostate-specific membrane antigen, show promising results, with a contrast superior to choline imaging and a higher detection rate even for low prostate-specific antigen levels; validation studies are ongoing. Finally, imaging targeting bone remodeling, using whole-body SPECT–CT, is a relevant complement to molecular/metabolic PET imaging when bone involvement is suspected.

## Introduction

Although radical prostatectomy (RP) with or without lymphadenectomy remains one of the main curative options for prostate cancer (PCa), more than 30% of the patients will relapse during follow-up (1). Salvage radiotherapy (SRT) represents the main treatment option for relapsing patients after RP, and durable biochemical response rates have been reported (2). Despite gains in understanding how to select patients for salvage treatment, the variable clinical course of these patients still leaves uncertainties about how and when to appropriately manage these patients.

Early identification of relapsing disease by modern imaging techniques has been demonstrated to significantly influence final treatment decisions and drive SRT in locally or locoregionally relapsing patients in terms of target volume definition as well as planned doses. Indeed, the effective dose necessary to achieve biochemical control in the SRT setting may be different if the tumor recurrence is micro- or macroscopic (3). At the same time, irradiation of only the prostatic bed or of the whole pelvis will depend on the precise location of the recurrence, local or loco-regional.

In the “theragnostic imaging” era, molecular imaging using positron emission tomography (PET) and single-photon emission computed tomography (SPECT) constitutes a useful tool for clinicians to define the site of the recurrence, the extent of disease, and allows, therefore, for individualizing salvage treatments. In the following review, we report on the evidence concerning the use of molecular imaging in the SRT setting in patients presenting with biochemical relapse after RP, with a special focus on new PCa-specific PET tracers. Table 1 provides a summary of the most relevant tracers available in the setting of post-prostatectomy relapsing PCa.

**Table 1:** **Summary of the most relevant tracers available for the evaluation of recurrent PCa**.

Tracer	Target	Technique	Use	Site of PCa recurrence	Main advantage	Main limitation
^18^F/^11^C-choline	Cell membrane synthesis and phospholipid metabolism	PET/CT PET/MR	Established	Any	Sensitivity	Lack of specificity for PCa
^18^F-NaF	Bone remodeling	PET/CT PET/MR	Established	Bone metastases	Sensitivity	Lack of specificity for PCa
^99m^Tc-diphosphonates	Bone remodeling	SPECT/CT	Established	Bone metastases	Sensitivity	Lack of specificity for PCa
^68^Ga-HBED-CC	PSMA	PET/CT PET/MR	Under evaluation	Any	Preliminary data showing higher sensitivity than choline-based tracers	To be assessed
^111^In–^111^In Capromab Pendetide (ProstaScint^®^)	PSMA	SPECT/CT	Established	Any	Specificity	Spatial resolution

*PCa, prostate cancer; PSMA, prostate-specific membrane antigen*.

## Evaluation of Local and Lymph Node Involvement Recurrence by Choline PET Tracers

^18^F-fluorodeoxyglucose (FDG) PET imaging is a well-established tool in radiation therapy planning, extensively used in many tumor types. The lack of FDG avidity in most PCa has motivated the search for alternative metabolic tracers, and among them, the most commonly used are choline tracers. Three main choline-based PET tracers exist, namely, ^11^C-choline, ^18^F-methylcholine, and ^18^F-ethylcholine: regardless of the slight chemical differences impacting overall distribution and the lack of formal comparative studies, available data suggest that their diagnostic performance is overall similar (4). ^11^C-acetate is another tracer, less commonly used in PCa, sharing with choline tracers a similar distribution, and being transformed to phosphatidylcholine after uptake (5). Studies have shown that performance is similar to ^18^F-choline (6).

The literature on the use of choline PET in recurrent PCa is vast but inhomogeneous, and for this reason, its use in recent guidelines is suggested but not established, yet. Two recent meta-analyses have tried to overcome this limitation, with encouraging and converging results when selecting studies with common inclusion criteria, protocols, and standard of reference (7, 8). Both analyses obtained pooled sensitivities and specificities above 85% in patients with biochemical recurrence. For local recurrence, in particular, the sensitivity was 61% and the specificity 97% (8).

Indeed, when assessing a biochemical recurrence of PCa after RP, it should be taken in account that the detection rates vary with prostate-specific antigen (PSA) levels when using choline-labeled tracers (9–11). Choline PET–CT has shown interesting results when assessing lymph node recurrences with PSA >1 ng/mL, with sensitivity of 90% and specificity of 100% in a per-patient analysis, and 67 and 96% in a per-region analysis, respectively (12). Below this level of PSA, the recurrence detection rate with choline-labeled tracers decreases, essentially because of the lack of ability for PET to detect small lesions (of a few millimeters), presenting with low metabolism due to the spatial resolution limit of the technique (9, 10, 13, 14). Nevertheless, the sensitivity of choline PET is still above 50% in patients with PSA <1 ng/mL when PSA doubling time is <6 months or PSA velocity is >1 ng/mL/year (10, 15, 16). When the 1 ng/mL threshold is not reached and other criteria, such as PSA doubling time and velocity, are not met, prostate-targeted magnetic resonance imaging (MRI) is considered the best choice to detect local recurrences. Conventional imaging, including CT and standard MRI, is, however, of limited value to identify metastatic lymph nodes since up to 80% of involved lymph nodes are smaller than 1 cm (17–19), and the evaluation of nodal involvement in prostate MRI studies is limited to the pelvic field of view. Integrated whole-body choline PET/MRI might thus be the modality of choice to overcome these limitations.

Choline PET–CT has been used to guide SRT planning, as recently reviewed (20). Despite the lack of large multicenter validation studies, single-center experiences consistently show that nodal and oligometastatic disease can be efficiently targeted (21–24). The limited spatial resolution remains the main obstacle for accurate targeting of the local relapse. Finally, more recent evidence has shown that choline PET also has a prognostic value among the candidates for curative radiation treatment (24, 25).

### The Added Value of Combined PET–MRI

**Figure 1:**
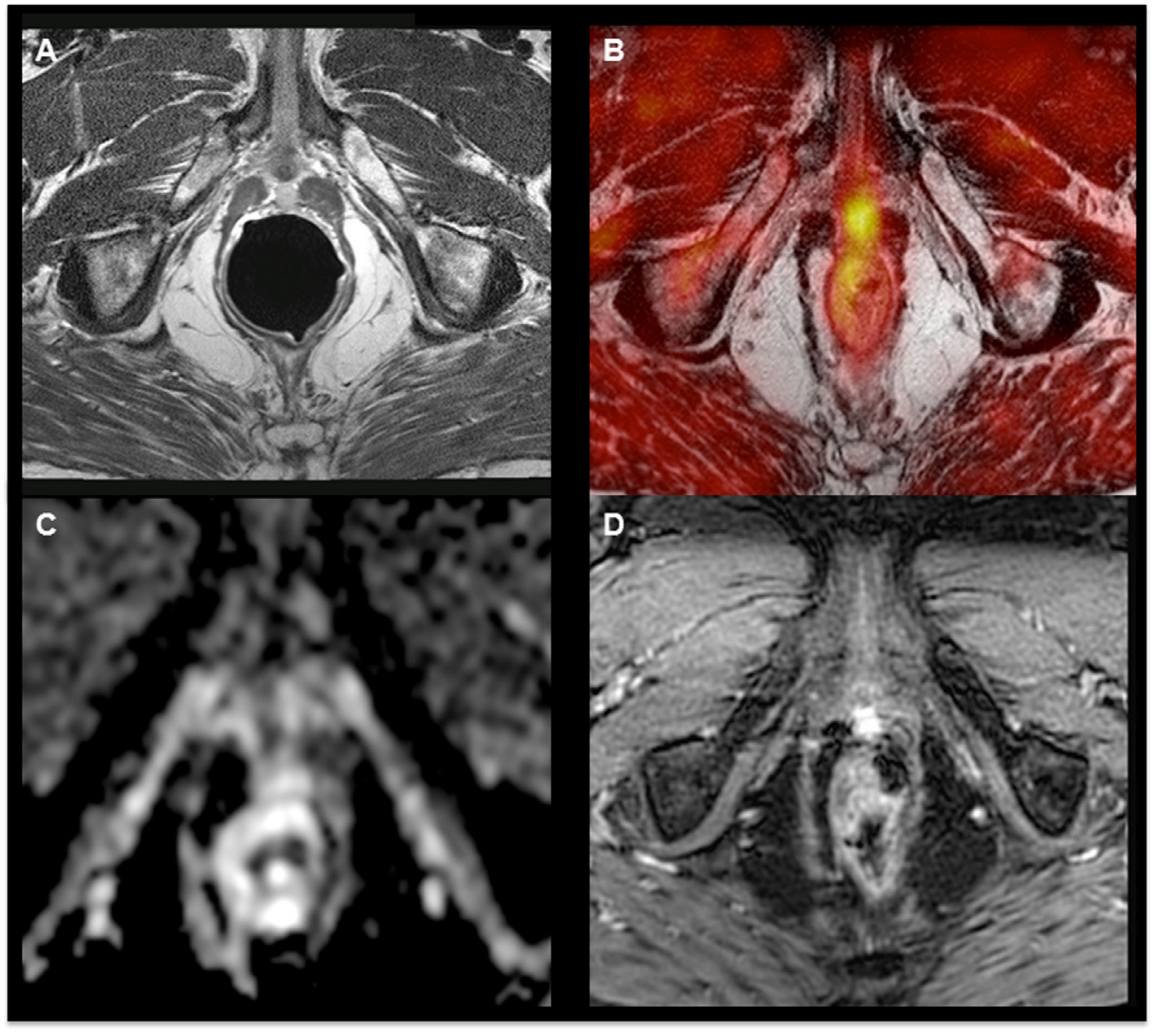
**^18^F-Fluorocholine hybrid PET–MRI images showing hyperintensity on the T2-weighted sequence (A) and focal hypermetabolism (B) in a nodule with limited diffusion restriction on ADC map (C) and hyperperfusion (D) in a patient with a biochemical relapse (PSA = 1.75 ng/mL, doubling time = 11 months) 9 years after radical prostatectomy**.

Magnetic resonance imaging is the most frequently used imaging modality to evaluate local PCa recurrence. T2-weighted imaging depicts recurrence with wide ranges of sensitivity and specificity with values of 48–100 and 50–100%, respectively, after RP and of 25–86 and 64–100%, respectively, after radiation therapy (26). Multiparametric imaging, such as spectroscopy, diffusion-weighted imaging, and dynamic contrast-enhanced MRI, have gained acceptance to complement T2-weighted MRI for primary and recurrent PCa detection (27–29). However, there is still an important need to further improve the accuracy of PCa imaging. The question arises whether associating metabolic PET data with MRI might potentially enhance PCa imaging. Preliminary reports using both modalities have provided contradictory results that could be explained in part by the difficulty to perform an accurate coregistration of the PET and MR images (30, 31). To solve this issue, hybrid PET–MRI systems have been designed to allow serial or simultaneous PET and MRI acquisitions during a single examination, with a common referential of the patient’s position. Acquiring fluorocholine PET and MRI in one single examination session showed a relevant improvement of the accuracy of PCa lesions’ detection (32–34) (Figure 1).

The adjunction of the PET acquisition leads to an important gain of the specificity of cancer detection when compared to MRI alone, without significant reduction of sensitivity for primary PCa staging. The sensitivity and specificity for the multiparametric MRI alone were 84.4 and 68.6%, respectively, and 81.2 and 87.1%, respectively, for the use of integrated PET–MRI (33). Another study showed that PCa was correctly detected in 80% of patients using ^18^F-choline PET alone, in 83.3% of patients using multiparametric MRI, and in 93.3% using integrated PET–MRI (34). These data show the ability of the PET–MRI scanner to perform MRI examinations of high diagnostic quality without artifacts related to the presence of the PET gantry and demonstrate that the information obtained from MRI (T2 anatomical sequences, diffusion, and perfusion) and PET (SUVmax) are complementary. Hitherto, no study has been published concerning the specific use of hybrid PET–MRI systems for recurrence detection or radiation therapy planning. However, there are ongoing studies scoping the development of dedicated positioning devices and dosimetric approaches (35, 36).

## Bone Metastases Assessment

Current guidelines recommend bone imaging only in selected high-risk cases. However, this definition is not homogenous in the literature (37, 38). In clinical practice, bone imaging is frequently performed in patients presenting with biochemical recurrence. Several choices exist, including bone scintigraphy, ^18^F-NaF PET–CT, or choline-labeled (^18^F or ^11^C) PET–CT (39).

Bone scintigraphy remains a widely used imaging modality in the metastatic workup of PCa patients. It allows for whole-body screening and is highly sensitive in the detection of metastases, but its specificity is limited due to benign conditions presenting also with altered tracer uptake (e.g., degenerative joint diseases, fractures, infections, or benign bone tumors) (40, 41). During the last decade, SPECT–CT has gained a wide acceptance for bone scanning. Many studies have shown that SPECT–CT reduces the rate of equivocal lesions compared to planar bone scan due to better anatomic localization of lesions and higher lesion-to-background contrast. By consequence, it increases diagnostic accuracy over SPECT alone or planar scintigraphy alone (42–46). Some authors use SPECT–CT only to clarify the origin of equivocal lesions based on planar scintigraphy, whereas others recommend to systematically acquire whole-body SPECT–CT from the cervical spine to the proximal femurs (43, 47). The proportion of indeterminate bone lesions can be reduced from a rate between 48 and 72% with planar whole-body scintigraphy and/or SPECT without CT, to a rate between 0 and 15% when adding SPECT with CT. Furthermore, SPECT–CT has been able to correctly convert a metastatic status into a non-metastatic status (downstaging) in 29.5% of the patients, with a sensitivity and specificity of 96.4 and 94.2%, respectively, on a per-patient analysis (47).

^18^F-NaF PET–CT is considered to have superior pharmacokinetic characteristics, such as high bone affinity, rapid clearance, and low protein binding, compared to ^99m^Tc-diphosphonates. Its impact in PCa management has been recently evaluated by the National Oncologic PET Registry (NOPR) in the US, showing a 44% rate of change in management in recurrent PCa (48). The patient-based analysis showed that sensitivity and specificity of ^18^F-fluoride PET–CT and bone scan were 96 versus 88% and 91 versus 80%, respectively (49). Although ^18^F-NaF PET–CT has been reported to be more sensitive for detection of metastases than planar bone scan, the question arose to know whether ^18^F-NaF PET–CT outperforms whole-body SPECT–CT. Indeed, the comparative studies available hitherto only compare ^18^F-NaF PET–CT to standalone SPECT acquisitions, which are intrinsically limited by the lack of anatomical correlation (50).

Radiolabeled choline PET–CT is used in the assessment of PCa recurrence in the prostate bed or in lymph nodes but can also highlight bone metastases (9, 14, 51). It has been reported that ^18^F-choline PET–CT was more specific than ^18^F-NaF PET–CT (99 versus 93%) but that ^18^F-choline PET–CT suffered from slightly lower sensitivity (74 versus 81%) (49, 52). There is still an uncertainty whether these choline-negative lesions could be a result of androgen-deprivation therapy, since many patients enrolled in trials are under androgen deprivation. Based on this finding, it is recommended to systematically carry out imaging reflecting bone remodeling (^18^F-NaF PET–CT or whole-body SPECT–CT) in addition to choline PET imaging for bone assessment, both for diagnostic and for treatment planning purposes, whenever bone involvement is suspected clinically.

## Future Tracers

While PET imaging currently validated for clinical practice is based on relatively unspecific tracers, such as FDG and choline, ongoing research focuses on the development of new tracers targeting tumor-specific antigens. The most promising tracers for prostate imaging are summarized below. No validation about their use in SRT is yet available, even if this has been tested for prostate-specific membrane antigen (PSMA) and anti-1-amino-3-^18^F-fluorocyclobutane-1-carboxylic acid (FACBC) tracers (53, 54).

### Prostate-Specific Membrane Antigen Tracers

Prostate-specific membrane antigen is a transmembrane protein overexpressed in PCa and highly expressed in androgen-independent disease (55). Preclinical and *in vitro* studies suggest a good specificity of this target when compared to normal prostatic tissue or post-radiation therapy fibrotic changes (56). The high specificity of this target has also motivated the development of therapeutic or combined diagnostic/therapeutic (or “theragnostic”) agents, radiolabeled with ^111^In or ^177^Lu (57, 58). PSMA imaging is performed using ^111^In Capromab Pendetide (ProstaScint^®^), a monoclonal murine antibody. This tracer is FDA approved for staging high-risk PCa and for recurrent PCa post-prostatectomy. Prostascint imaging has, however, some disadvantages: a complex biodistribution, requiring imaging up to 6 days after administration, an intracellular epitope, not accessible in living cells, non-specific signal in the presence of inflammation, and the intrinsic lower resolution of SPECT imaging as compared with PET (59).

A comprehensive description of all tracers developed in preclinical studies for this target goes beyond the scope of this paper. Therefore, we will only briefly summarize the results of the clinical studies performed so far in recurrent PCa. Four tracers have been used in human studies, three of them using ^18^F as radioisotope and one using ^68^Ga.

#### ^18^F-DCFBC

A dosimetry study in five metastatic patients showed the ability of the tracer to detect probable metastatic lesions in lymph nodes and the skeleton (60). The tracer has also been evaluated in primary PCa cancer characterization in 13 patients, showing a high specificity for tumor lesions over benign hypertrophy, even higher than MRI (61).

#### ^18^F-BAY1075553

Only a single phase I study has been published, including 12 patients (9 at staging and 3 with recurrent PCa), and comparing the diagnostic performance of this tracer to ^18^F-choline, showing a similar performance of the two tracers for the characterization of prostatic lesions. However, ^18^F-choline has been shown to be superior for nodal and bone marrow lesions’ detection (62).

#### ^18^F-DCFPyL

Only two studies used this tracer in patients, one of them performing whole-body dosimetry and the other providing a preliminary comparison with ^68^Ga-HBED-CC in 14 patients with recurrent PCa (63, 64).

#### ^68^Ga-HBED-CC

This is the most extensively evaluated PSMA tracer so far, with already over 20 published studies. All of them showed high proportions of positive findings in recurrent disease, with detections rates ranging from 82.8 to 89.5%, in the two largest studies (65, 66). In patients with PSA values between 0.2 and 0.5 ng/mL, the detection rate was 57.9% (66). One study suggests superiority in comparison with ^18^F-choline, with higher contrast and more lesions identified by the PSMA marker (67). Discordant results were found with respect to the impact of PSA doubling time on PET positivity (66, 68). Only one recent study has evaluated the impact of this tracer on radiation therapy planning, showing a change in strategy in about 50% of the cases, which is in line with the range of the management changes rate reported for choline (54, 69, 70).

### Amino Acids

Amino acid demand and transport are increased in malignant prostatic cells, reflecting protein synthesis. Some radiolabeled amino acids have been developed in order to explore this metabolic pathway. Anti-(^18^F)-FACBC (anti-1-amino-3-^18^F-FACBC or fluciclovine) appears to be a promising PET amino-acid radiotracer: it is a synthetic l-leucine analog, leucine being an essential nutrient for protein synthesis and cell growth, with high uptake in the majority of PCa lesions and metastasis. In a recent meta-analysis of six studies concerning the performances of ^18^F-FACBC PET–CT in patients with a suspicion of PCa recurrence, the pooled sensitivity and specificity for this radiotracer were 87 and 66%, respectively (71). Comparative studies with choline tracers showed a higher sensitivity and specificity, with an approximately 20% higher detection rate when using ^18^F-FACBC (72–75).

### Gastrin-Releasing Peptide Receptors

Gastrin-releasing peptide receptors (GRPR) are overexpressed in a majority of PCa cells. Therefore, they represent a potential target for diagnostic imaging procedures. Bombesin, which can be labeled with positron-emitting radionuclides, is one of those tracers. Different radiolabeled bombesin analogs have been tested in primary and metastatic PCa (76, 77) as well as in cases of biological recurrence after surgery or hormonal therapy (76). Kähkönen et al., using ^68^Ga-labeledDOTA-4-amino-1-carboxymethyl-piperidine-d-Phe–Gln–Trp–Ala–Val–Gly–His–Sta–Leu-NH2 peptide (BAY 86-7548), found satisfying results in detection of recurrence in prostatic bed and nodal relapse but poor ability to detect bone metastases (76). Sah et al. published a first-in-man study concerning BAY 864367, a slightly different ^18^F-labeled bombesin tracer (78). They found that the tracer uptake was higher in primary PCa than in recurrent lesions. Mitsakis et al. compared ^68^Ga-NODAGA-MJ9 (MJ9) PET–CT with ^18^F-flurocholine in 33 patients with recurrent PCa and concluded that MJ9 missed 75% of the 24 bone lesions identified on ^18^F-choline PET. However, 18% of metastatic lymph nodes that were positive on 18-flurocholine were negative on MJ9, and inversely, 13% of lesions in lymph nodes were positive on MJ9 but negative on ^18^F-flurocholine PET/CT, with a greater signal-to-background ratio on MJ9 images (79).

### Fluoro-5-Dihydrotestosterone

16β-(^18^F)-fluoro-5-dihydrotestosterone (FDHT) is a fluorinated testosterone analog that can detect the overexpression of androgen receptors in PCa lesions. The first study concerning the use of FDHT in patients with progressive metastatic PCa showed a high tumor-to-background ratio and a detection rate of 78% of the 59 lesions identified on conventional imaging methods in a group of seven patients (80). Tumor uptake of FDHT is receptor mediated (81), and thus, the results of the FDHT–PET may be able to predict which lesions will show a good response to androgen deprivation therapy and which ones will not, therefore, needing another type of treatment (82). Moreover, the intensity of FDHT uptake in bone metastases of castration-resistant PCa patients was a negative prognostic factor in terms of patient survival (83). No studies on the use of FDHT in recurrent PCa after RP have been published, yet.

## Conclusion

The combination of radiolabeled-choline PET and MRI appears to be the modality of choice in clinical routine for the assessment of recurrence of PCa, associating the identification of nodal and distant disease based on PET and the local assessment by multiparametric MRI. While the availability of integrated PET–MRI systems will presumably remain confined to academic centers, at least in the near future, the use of software allowing automated fusion of PET and MRI sequences acquired at different times is already widely used in SRT planning. A new generation of targeted tracers, such as PSMA and FACBC, has shown promising results, with a lesion-to-background contrast superior to choline imaging and a higher detection rate of lesions even for very low PSA levels. Results of ongoing validation studies are warranted. Bone remodeling tracers, including standard bone scans with SPECT–CT, remain of great interest in assessment of bone extension and should be systematically associated with metabolic imaging.

## Author Contributions

TZ, VG, ORager, and GA are responsible for the study design and contributed equally to the manuscript. TZ, VG, ORager, GA, and CT-V drafted the manuscript. RM, GG, ORatib, TP, and ES revised the manuscript. All authors read and approved the final manuscript.

## Conflict of Interest Statement

The authors declare that the research was conducted in the absence of any commercial or financial relationships that could be construed as a potential conflict of interest.
